# Supplementary material for: Argon Adsorption on Cationic Gold Clusters Aun+ (n ≤ 20)
Source: Molecules. 2021 Jul 4;26(13):4082. doi: 10.3390/molecules26134082 (PMC8272223; doi:10.3390/molecules26134082)
Supplement: Supplementary file 1 [file molecules-26-04082-s001.zip › molecules-1275456-supplementary.pdf]

Supporting Information

Argon adsorption on cationic gold clusters Au_n^+ ($n \leq 20$)

Piero Ferrari [1] and Ewald Janssens [1,*]

[1]Quantum Solid-State Physics, Department of Physics and Astronomy, KU Leuven, Leuven, Belgium

*Correspondence: ewald.janssens@kuleuven.be

1. Ar adsorption site isomers of Au_4Ar^+ and Au_6Ar^+

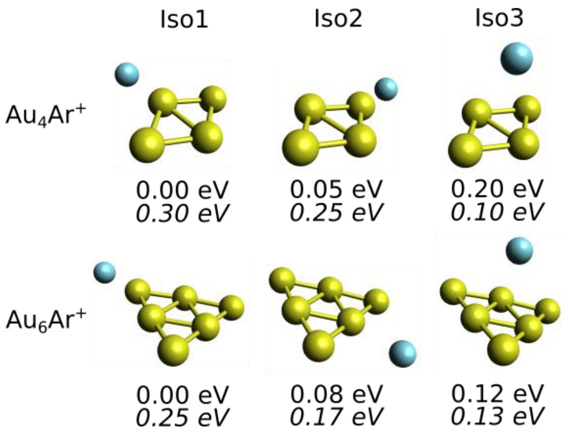

Figure S1. Ar adsorption site isomers of Au_4Ar^+ and Au_6Ar^+ clusters. The energies relative to the lowest energy adsorption site are presented below each structure, as well as the Ar adsorption energy (E_{ads}, in italics) of each isomer.

2. Optimized geometries of $Au_nAr_m^+$ (n = 3 – 20; m = 1 - 5) clusters

The coordinates of those clusters are available through Github at *https://github.com/pferrari13/Au_nAr_m-XYZ-coordinates.git*

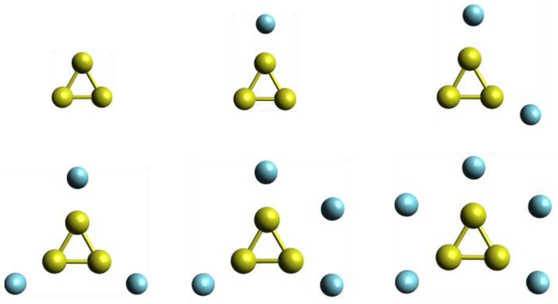

Figure S2. Optimized geometries of $Au_3Ar_m^+$ (m = 0-5).

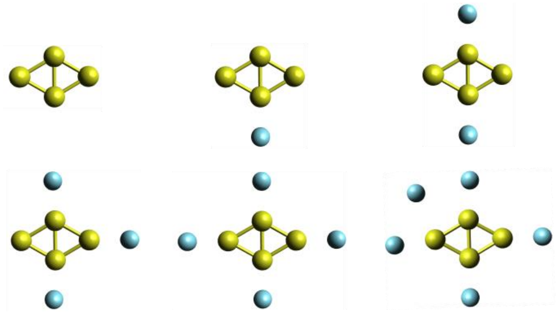

Figure S3. Optimized geometries of $Au_4Ar_m^+$ (m = 0-5).

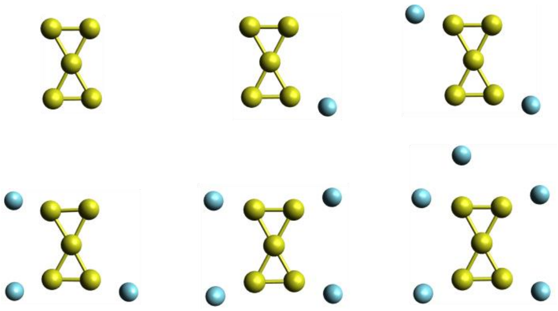

Figure S4. Optimized geometries of $Au_5Ar_m^+$ (m = 0-5).

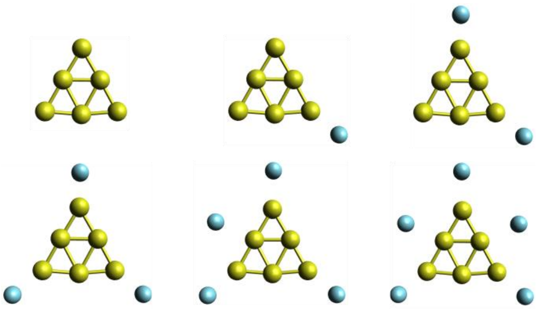

Figure S5. Optimized geometries of $Au_6Ar_m^+$ (m = 0-5).

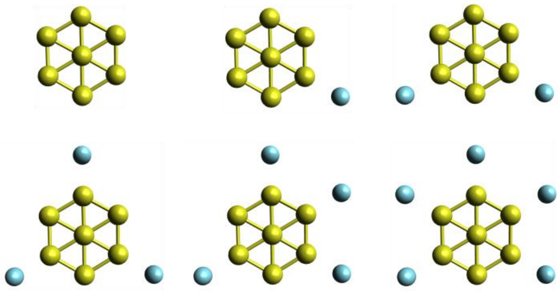

Figure S6. Optimized geometries of $Au_7Ar_m^+$ (m = 0-5).

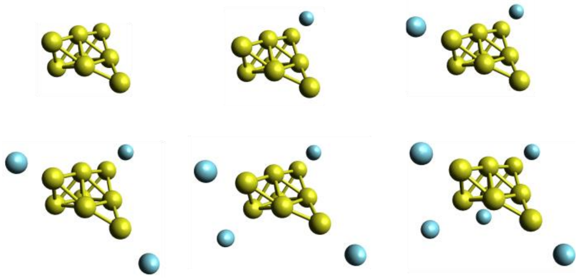

Figure S7. Optimized geometries of $Au_8Ar_m^+$ (m = 0-5).

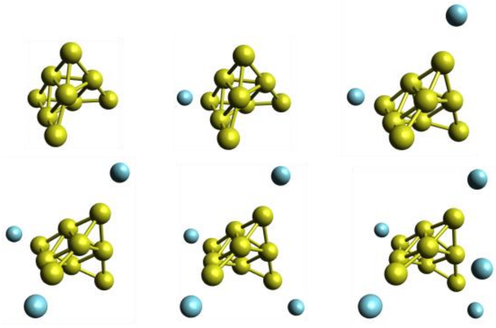

Figure S8. Optimized geometries of $Au_9Ar_m^+$ (m = 0-5).

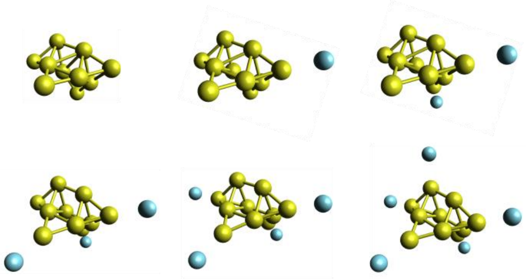

Figure S9. Optimized geometries of $Au_{10}Ar_m^+$ (m = 0-5).

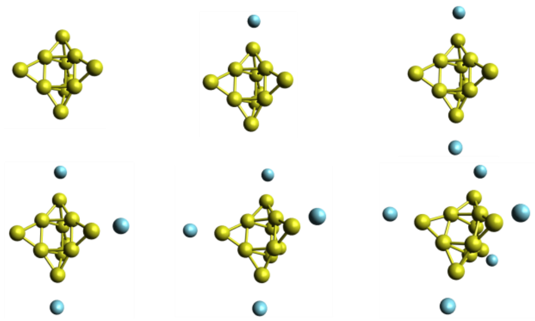

Figure S10. Optimized geometries of $Au_{11}Ar_m^+$ (m = 0-5).

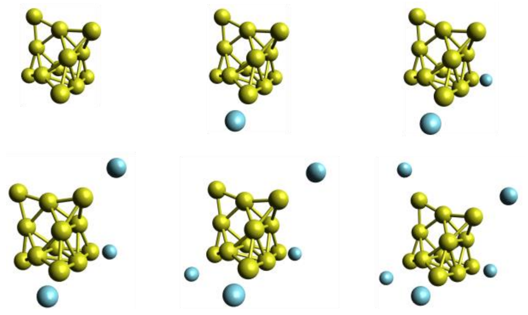

Figure S11. Optimized geometries of $Au_{12}Ar_m^+$ (m = 0-5).

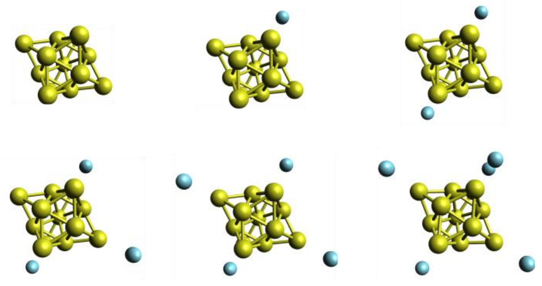

Figure S12. Optimized geometries of $Au_{13}Ar_m^+$ (m = 0-5).

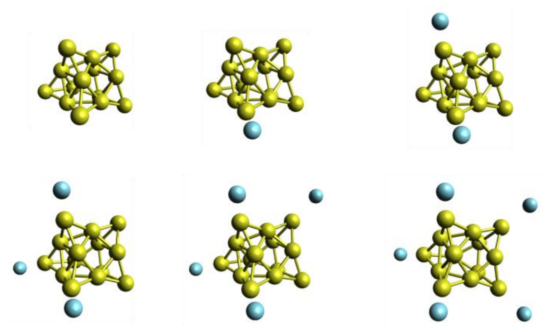

Figure S13. Optimized geometries of $Au_{14}Ar_m^+$ (m = 0-5).

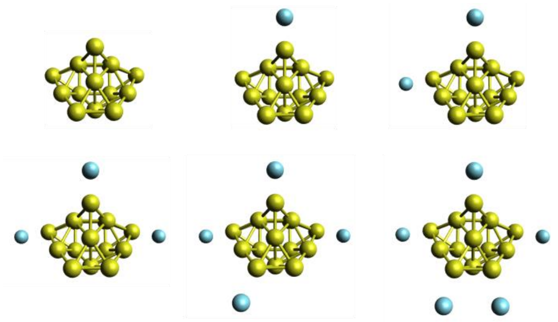

Figure S14. Optimized geometries of $Au_{15}Ar_m^+$ (m = 0-5).

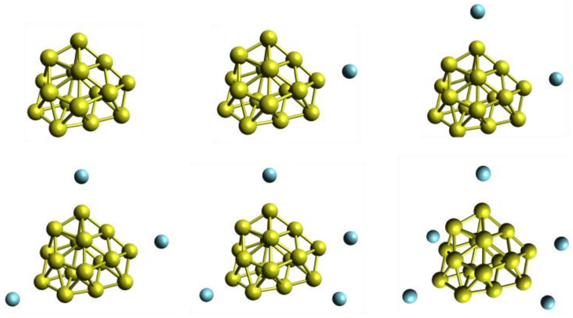

Figure S15. Optimized geometries of $Au_{16}Ar_m^+$ (m = 0-5).

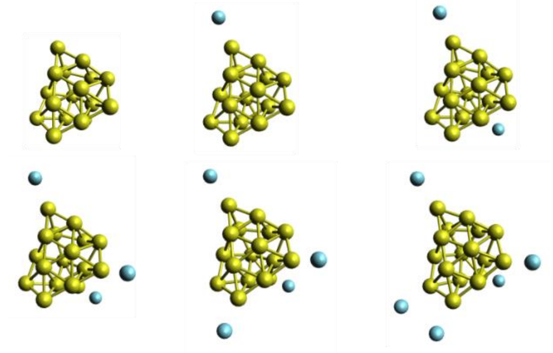

Figure S16. Optimized geometries of $Au_{17}Ar_m^+$ (m = 0-5).

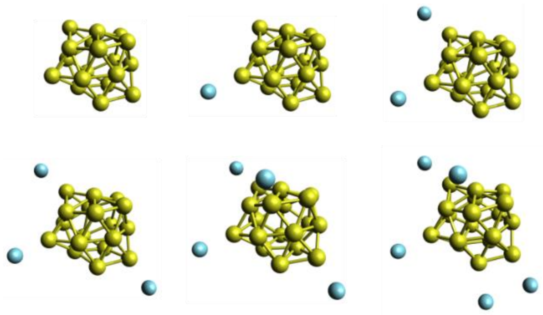

Figure S17. Optimized geometries of $Au_{18}Ar_m^+$ (m = 0-5).

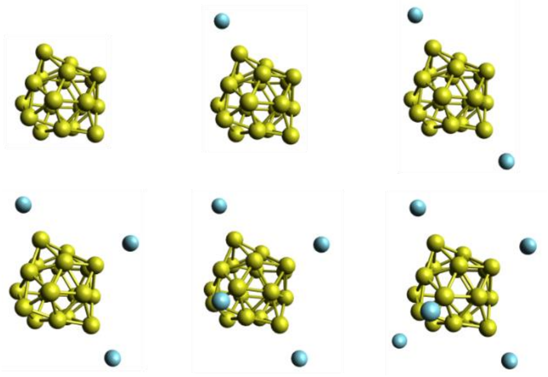

Figure S18. Optimized geometries of $Au_{19}Ar_m^+$ (m = 0-5).

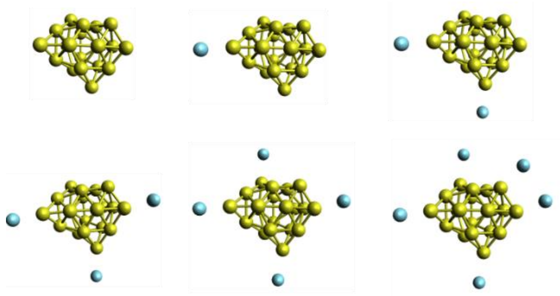

Figure S19. Optimized geometries of $Au_{20}Ar_m^+$ (m = 0-5).
